# Modulation of Macrophage Inflammatory Responses by UDP-Glucuronosyltransferase-Mediated PGE_2_ Glucuronidation

**DOI:** 10.3390/jpm16030160

**Published:** 2026-03-13

**Authors:** Dahye Lee, Hee Young Cho, Sangzin Ahn, Yong-Soon Cho, Dong Hyun Kim, Jae-Gook Shin, Su-Jun Lee

**Affiliations:** 1 Department of Pharmacology and PharmacoGenomics Research Center, Inje University College of Medicine, Inje University, Busan 47392, Republic of Korea; ddahye0620@gmail.com (D.L.); hee278@inje.ac.kr (H.Y.C.); sangzinahn@inje.ac.kr (S.A.); ysncho@gmail.com (Y.-S.C.); dhkim@inje.ac.kr (D.H.K.); phshinjg@gmail.com (J.-G.S.); 2 Center for Personalized Precision Medicine of Tuberculosis, Inje University College of Medicine, Inje University, Busan 47392, Republic of Korea

**Keywords:** macrophages, UGTs, glucuronidation, PGE_2_, inflammation

## Abstract

**Background/Objectives:** Macrophages polarized into M1 and M2 phenotypes differentially regulate immune and drug responses. Despite their distinct functional roles, differences in UDP-glucuronosyltransferase (UGT) expression and enzymatic activity between M1 and M2 macrophages remain poorly understood. This study aimed to characterize differential UGT expression in M1 and M2 macrophages and to elucidate how UGT-mediated prostaglandin E_2_ (PGE_2_) glucuronidation modulates macrophage inflammatory responses. **Methods:** THP-1 cells were chemically differentiated into macrophages (M0) and subsequently polarized into M1 and M2 phenotypes. UGT expression profiles were assessed using RT-PCR, quantitative RT-PCR (qRT-PCR), and Western blot. UGT activity was compared by quantifying glucuronide metabolites derived from UGT-specific substrates using LC-MS/MS, along with measurement of free PGE_2_ and PGE_2_-glucuronide by ELISA. Pro-inflammatory cytokine expression and secretion in M1 macrophages were quantified using qRT-PCR and ELISA. **Results:** Expression of UGT1A1, UGT1A4, UGT1A5, UGT1A9, and UGT2B7 were markedly higher in M1 compared with M2 macrophages at both the mRNA and protein levels. Enhanced UGT activity in M1 macrophages was reflected by increased formation of estradiol-3-glucuronide and naloxone-3-glucuronide (both *p* < 0.01) and was attenuated in a concentration-dependent manner by diclofenac. Furthermore, PGE_2_ glucuronidation was more pronounced in M1 macrophages, and inhibition of UGTs with atazanavir reduced PGE_2_-glucuronide formation and pro-inflammatory cytokine production, including IL-1β, IL-6, and TNF-α. **Conclusion:** UGT-mediated PGE_2_ glucuronidation in M1 macrophages contributes to the regulation of pro-inflammatory cytokine production. Collectively, these findings support a role for UGTs as modulators of inflammatory signaling, with differential expression and activity between M1 and M2 macrophages.

## 1. Introduction

Macrophages are fundamental components of the innate immune system and play critical roles in host defense and immune regulation [1]. Among their heterogeneous populations, M1 and M2 macrophages are recognized as the two predominant phenotypes, each exhibiting distinct functional characteristics [2]. M1 macrophages are classically activated cells characterized by potent pro-inflammatory and anti-microbial functions, contributing to the initiation and amplification of immune responses during infection and tissue injury [3]. In contrast, M2 macrophages are alternatively activated and primarily exert anti-inflammatory effects, facilitating resolution of inflammation, tissue repair, and wound healing [4]. The dynamic balance between M1 and M2 macrophages is crucial for maintaining immune homeostasis, and its dysregulation is implicated in various pathological conditions, including chronic inflammatory diseases and tumor progression [5].

Despite extensive research on macrophage polarization, the role of UDP-glucuronosyltransferases (UGTs) in macrophage biology remains largely unexplored. UGTs are phase II metabolic enzymes predominantly expressed in the liver and are responsible for the detoxification and elimination of a broad spectrum of xenobiotics through glucuronidation [6]. In addition to drug metabolism, UGTs also regulate endogenous substrates, including lipid mediators and steroid hormones, thereby influencing cellular signaling pathways and physiological functions [7]. Although UGT expression and activity have been well characterized in hepatic tissues [8,9], their expression profiles and functional relevance in M1 and M2 macrophages are not well defined.

Prostaglandin E_2_ (PGE_2_) is a key lipid mediator that modulates immune and inflammatory responses in macrophages [10]. PGE_2_ promotes vasodilation during the early phase of inflammation and regulates the activity of various immune cells, including neutrophils, macrophages, dendritic cells, and mast cells [11]. It enhances the production of the anti-inflammatory cytokine interleukin-10 (IL-10) while suppressing pro-inflammatory cytokines such as IL-1β, IL-6, and tumor necrosis factor alpha (TNF-α) [12]. Although pharmacological strategies targeting PGE_2_ synthesis, such as corticosteroids and nonsteroidal anti-inflammatory drugs (NSAIDs), are widely used, their adverse effects and limited efficacy in chronic inflammation underscore the need for alternative regulatory mechanisms [13]. Notably, UGTs can inactivate PGE_2_ through glucuronidation, thereby reducing its bioavailability [14]. Given that UGT-mediated metabolism modulates intracellular levels of bioactive lipids and hormones [7], glucuronidation of PGE_2_ may represent an important mechanism controlling macrophage inflammatory function.

Therefore, this study aimed to investigate UGT expression patterns in M1 and M2 macrophages and to elucidate the role of UGT-mediated PGE_2_ glucuronidation in regulating inflammatory cytokine responses. Our findings suggest that macrophage UGTs may serve as novel modulators of inflammation and potential therapeutic targets in inflammatory diseases.

## 2. Materials and Methods

### 2.1. Chemicals and Reagents

All chemicals and reagents were procured from Sigma-Aldrich (St. Louis, MO, USA) unless otherwise stated. Phorbol 12-myristate 13-acetate (PMA) was used to differentiate THP-1 cells into M0 macrophages. Lipopolysaccharide (LPS) and human interferon gamma (IFN-γ) were utilized to polarize M0 into M1 macrophages. Recombinant humanIL-4 and IL-13 were employed for M2 macrophage polarization. Diclofenac and atazanavir (ATZ) served as the UGT inhibitor in our experiments.

### 2.2. Macrophage Culture, Differentiation and Polarization

The culture of macrophages and their polarization into M1 and M2 cells were performed using methods previously established in our laboratory [15]. Briefly, THP-1 cells were maintained in RPMI 1640 medium supplemented with 10% fetal bovine serum (FBS), 2-mercaptoethanol (0.05 mM) and 1% penicillin-streptomycin. For differentiation into M0 macrophages, THP-1 cells were treated with PMA (200 ng/mL) for 24 h. To further polarize into M1, M0 macrophages were exposed to LPS (100 ng/mL) and human IFN-γ (20 ng/mL) for 24 h. For M2 polarization, M0 macrophages were treated with recombinant human IL-4 and IL-13 (both at 20 ng/mL) for 24 h.

### 2.3. Reverse Transcription Polymerase Chain Reaction (RT-PCR) and Quantitative Real-Time PCR (qRT-PCR)

Total RNA was isolated from the cells using TRIzol reagent (Invitrogen, Carlsbad, CA, USA). Approximately 1 μg of RNA was reverse transcribed using the iScript cDNA Synthesis Kit (Bio-Rad Laboratories, Hercules, CA, USA). RT-PCR was first performed to determine which UGT genes were expressed among all UGT isoforms. Synthesized cDNA was subjected to PCR amplification in a 50 μL reaction volume containing 10 mM dNTPs, 25 mM MgCl_2_, and 10 pmol of both the forward and reverse primers (Supplementary Table S1). qRT-PCR was then performed with CFX96 Touch Real-Time PCR Detection System (Bio-Rad Laboratories) using TaqMan probe sets for GAPDH (catalogue number Hs02786624_m1), UGT1A1 (catalogue number Hs00426380_m1), UGT 1A4 (catalogue number Hs01655285_s1), UGT1A5 (catalogue number Hs01374521_s1), UGT1A9 (catalogue number Hs02516855_s1), UGT2B7 (catalogue number Hs00426592_m1), IL-1β (catalogue number Hs01555410_m1), IL-6 (catalogue number Hs00174131_m1), and TNF-α (catalogue number Hs00174128_m1) acquired from Thermo Fisher Scientific (Waltham, MA, USA). The relative expression of genes was normalized to GAPDH using the 2^−ΔΔCt^ method [16].

### 2.4. Western Blot

Cells were lysed using RIPA buffer supplemented with protease and phosphatase inhibitors. Protein concentrations were measured using the BCA Protein Assay Kit (Pierce Biotechnology, Rockford, IL, USA). Samples were then resolved on SDS-PAGE and transferred to nitrocellulose membranes. Membranes were blocked with 5% BSA and incubated overnight at 4°C. Primary antibodies against UGT1A1, UGT1A4, UGT1A9, and UGT2B7 were used at a dilution of 1:1000 (Abcam, Cambridge, UK). After washing, membranes were incubated with HRP-conjugated secondary antibodies. Bands were visualized using ECL detection system and images were captured using a ChemiDoc Imaging System (Bio-Rad Laboratories).

### 2.5. UGT Activity Assay Using Fluorescence-Based Detection System

After harvesting M1 and M2 macrophages, membrane fractions were extracted according to previously described methods [15]. UGT activity was measured using a fluorescence-based UGT activity assay according to the manufacturer’s instructions (Abcam). Briefly, membrane protein (500 μg) was added to the appropriate wells of a black 96-well plate, followed by the addition of reaction mixes including UGT substrates and assay buffer. The reaction was initiated by the addition of UDP-glucuronic acid (UDPGA). Fluorescence was immediately measured in kinetic mode for 40 min at 37 °C with excitation and emission wavelengths set at 415 and 502 nm, respectively (Molecular Devices, San Jose, CA, USA).

### 2.6. UGT Activity Assay Using LC/MS-MS

Selective enzymatic activities of UGT1A1 and UGT2B7 were measured independently using β-estradiol [17] and naloxone [18], respectively, as isoform-specific substrates in microsomes prepared from harvested cells. Reactions were initiated by the addition of NADPH and UDPGA and reaction mixture were analyzed by LC-MS/MS after 1 h of incubation using an API 5500 system (Applied Biosystems, Foster City, CA, USA) coupled to an Agilent 1290 high-performance liquid chromatography system. Estradiol-3-glucuronide was detected in negative ion mode using the transition *m*/*z* 447.0→271.0, with estrone glucuronide (*m*/*z* 445.0→269.0) used as the internal standard. Naloxone-3-glucuronide was detected in positive ion mode by monitoring the transition *m*/*z* 504.0→310.0 as previously described [19]. Peak areas were automatically integrated using Analyst software (version 1.4).

### 2.7. ELISA Assay

Cells were treated with arachidonic acid (ARA) in the presence or absence of ATZ, and culture media were collected at 3, 6, and 24 h after treatment for subsequent analyses. Concentration of PGE_2_ in culture media were quantified using a Prostaglandin E_2_ Parameter Assay Kit (R&D Systems, Minneapolis, MN, USA) according to the manufacturer’s instructions [20]. For the quantification of PGE_2_-glucuronide, samples were enzymatically digested with β-glucuronidase. Levels of IL-1β, IL-6, and TNF-α were quantified using Human Quantikine ELISA Kit (R&D Systems) in accordance with the manufacturer’s instructions [21].

### 2.8. Statistical Analysis

All experiments were independently performed three times, each including technical triplicate. Data are presented as mean ± standard deviation from one representative experiment. Comparisons between two groups were conducted using Student’s *t*-test, while comparisons among multiple groups were conducted one-way ANOVA followed by Tukey’s post hoc test. Statistical significance is indicated as * *p* < 0.05, ** *p* < 0.01, and *** *p* < 0.001.

## 3. Results

### 3.1. UGT Expression Profiles

**Figure 1:**
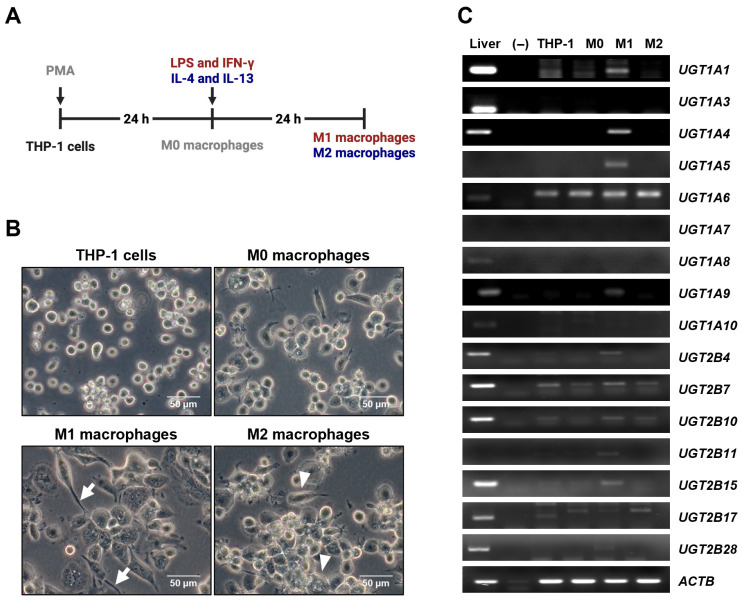
UGT expression profiling across THP-1-derived macrophages. (**A**) Experimental workflow for differentiation and polarization of THP-1 cells into M0, M1, and M2 macrophages. THP-1 cells were differentiated into M0 macrophages by PMA treatment and subsequently polarized into M1 using LPS and IFN-γ or into M2 macrophages using IL-4 and IL-13. (**B**) Representative phase-contrast images showing morphological changes during differentiation and polarization of THP-cells. M1 macrophages exhibit an elongated and sharpened morphology (arrows), whereas M2 macrophages display a flattened morphology (arrowheads). (**C**) Gene expression profiles of UGT isoforms in liver tissue, THP-1 cells, M0, M1, and M2 macrophages. The results presented are representative of one out of the three independent experiments. *ACTB* was used as a loading control. Liver tissue cDNA was used as a positive control for the detection of UGTs in the expected size of amplification. No bands were detected in negative control samples in which PCR was performed without reverse transcriptase.

THP-1 cells were cultured under optimized conditions and subsequently differentiated into diverse macrophage phenotypes upon appropriate stimulation (Figure 1A). Specifically, THP-1 cells were differentiated into a macrophage phenotype (M0) through PMA treatment as previously described [22]. The M0 macrophages were then polarized into M1 macrophages upon exposure to LPS and human IFN-γ [23], evident from the sharpened cell morphology. On the other hand, treatment with recombinant human IL-4 and IL-13 transformed M0 into M2 macrophages [24], characterized by their flattened shape (Figure 1B). To determine whether macrophage differentiation and polarization alter UGT expression, RT-PCR was performed in THP-1 cells, M0, M1, and M2 macrophages. Higher UGT mRNA expressions were observed in M1 compared with other cell types examined in this analysis, particularly for UGT1A1, UGT1A4, UGT1A5, UGT1A9, UGT2B4, UGT2B10, and UGT2B15 (Figure 1C), indicating distinct UGT mRNA expression patterns across macrophage types.

**Figure 2:**
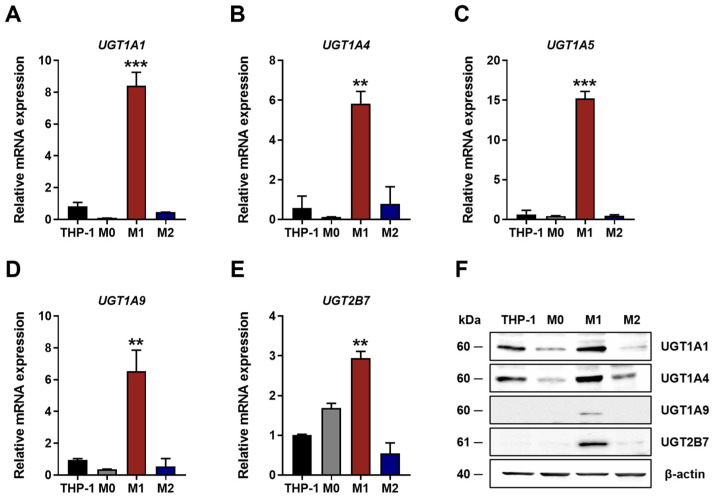
Predominant expression of UGT mRNAs and proteins in M1 macrophages. (**A**–**E**) Relative UGT mRNA expression in THP-1 cells, M0, M1, and M2 macrophages. Data are presented as fold changes in mRNA expression normalized to *GAPDH*. Values represent the mean ± SD of three independent experiments. ** *p* < 0.01, and *** *p* < 0.001 compared with THP-1. (**F**) Relative protein expression in THP-1 cells, M0, M1, and M2 macrophages, with β-actin used as loading control. The results presented are representative of one out of the three independent experiments.

Based on these results, UGT isoforms showing the greatest differences between M1 and M2 macrophages were selected for further validation. qRT-PCR analysis confirmed that mRNA expression levels of UGT1A1, UGT1A4, UGT1A5, UGT1A9, and UGT2B7 were significantly higher in M1 compared with M2 macrophages (Figure 2A–E). Consistent with these results, Western blot analysis showed increased protein expression of UGT1A1, UGT1A4, UGT1A9, and UGT2B7 in M1 macrophages, indicating that macrophage polarization is associated with differential regulation of UGT expressions.

### 3.2. UGT Enzymatic Activity in M1 and M2 Macrophages

**Figure 3:**
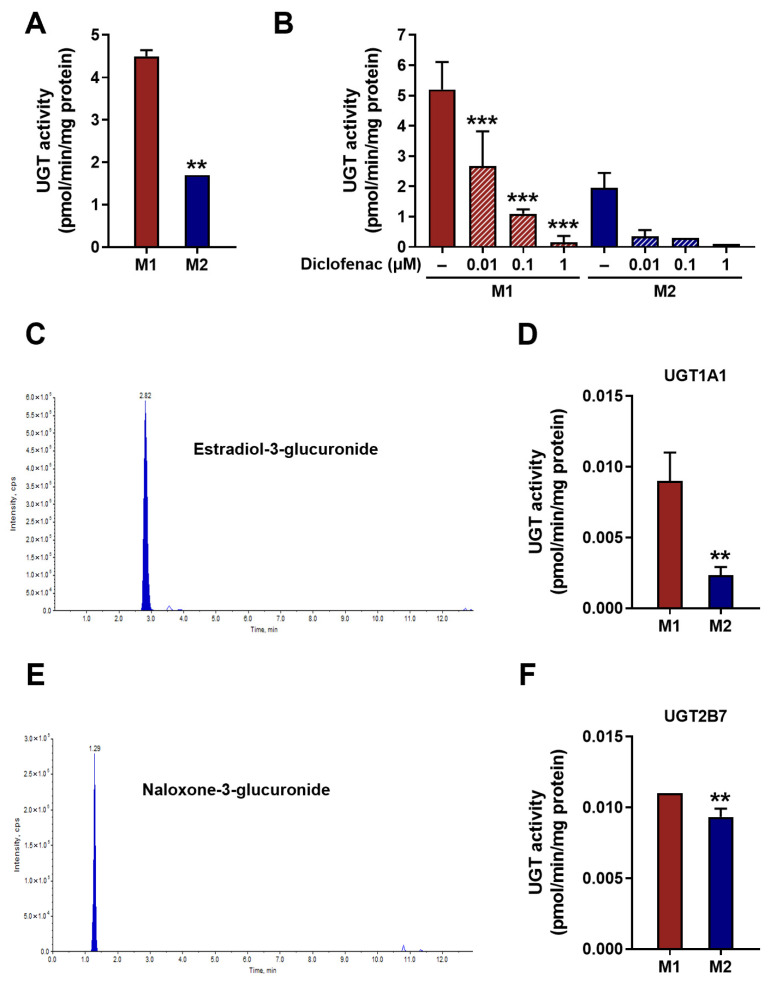
Strong UGT enzymatic activity in M1 compared with M2 macrophages. (**A**) Total UGT enzymatic activity measured in M1 and M2 macrophages. (**B**) Concentration-dependent inhibition of UGTs in M1 and M2 macrophages following treatment with diclofenac. Cross-hatched bars indicate the diclofenac-treated conditions. (**C**) Representative LC-MS/MS chromatogram showing the detection of estradiol-3-glucuronide as a UGT1A1-mediated metabolite. (**D**) UGT1A1-specific enzymatic activity in M1 and M2 macrophages. (**E**) Representative LC-MS/MS chromatogram showing the detection of naloxone-3-glucuronide as a UGT2B7-mediated metabolite. (**F**) UGT2B7-specific enzymatic activity in M1 and M2 macrophages. UGT activity was determined using total cellular protein extracts. Data are presented as the mean ± SD of three independent experiments. ** *p* < 0.01, and *** *p* < 0.001 compared with M1 or no treatment.

Following confirmation of differential UGT expression at both the mRNA and protein levels across macrophage phenotypes, enzymatic activity was subsequently evaluated to determine whether these expression differences translate into altered catalytic capacity. Beyond expression levels, differences in UGT activity between M1 and M2 macrophages were analyzed. In the assay using a fluorescent substrate for UGTs, it was observed that M1 macrophages had significantly higher UGT activity compared with M2 counterparts (*p* < 0.01) (Figure 3A). To ensure the specificity of UGT activity, a known UGT inhibitor, diclofenac was treated in the catalytic reaction [25]. As anticipated, the UGT activity reduced in a concentration-dependent manner in the presence of diclofenac (*p* < 0.001) (Figure 3B). To further probe the functional implications of UGT expression differences in these macrophages, the formation of glucuronidated metabolites produced by specific UGT isoforms were analyzed using LC-MS/MS as described previously [19]. Estradiol-3-glucuronide, a metabolite specifically produced by UGT1A1 [17] (Figure 3C) were examined in M1 and M2 macrophages. Comparison of estradiol-3-glucuronide production showed a 4-fold difference in metabolic capacity in M1 compared with M2 macrophages (*p* < 0.01) (Figure 3D). The formation of the UGT2B7-selective metabolite naloxone-3-glucuronide (Figure 3E) [18] was also significantly higher in M1 compared with M2 macrophages (*p* < 0.05) (Figure 3F). However, the difference in activity of UGT2B7 between M1 and M2 macrophages was not markedly greater than that of UGT1A1. Overall, higher UGT activity was consistently observed in M1 macrophages, accompanied by predominant UGT expression patterns.

### 3.3. Impact of UGT-Mediated PGE_2_ Glucuronidation in M1 Macrophages

**Figure 4:**
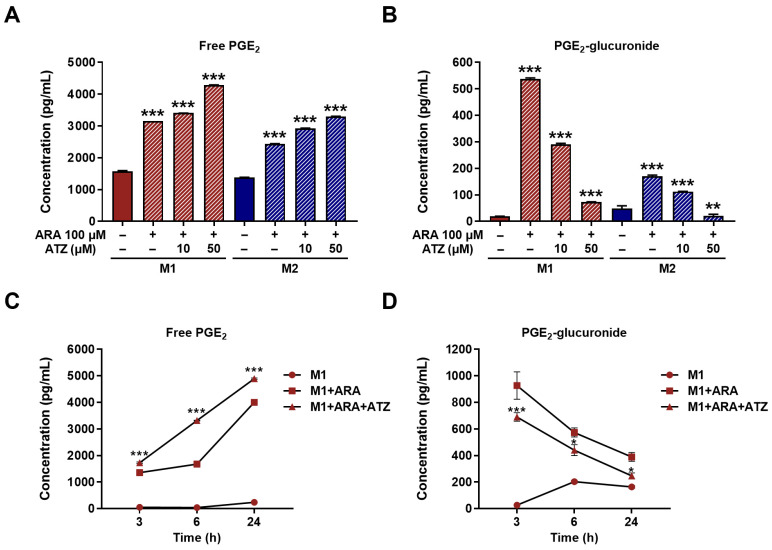
Influence of ATZ as UGT inhibitor on production of PGE_2_-glucuronide in M1 and M2 macrophages. Levels of (**A**) free PGE_2_ and (**B**) PGE_2_-glucuronide in M1 and M2 macrophages treated with 100 μM ARA in the presence or absence of the UGT inhibitor ATZ. Time-dependent changes in (**C**) free PGE_2_ and (**D**) PGE_2_-glucuronide levels in M1 macrophages treated with ARA alone or in combination with ATZ for the indicated times. Cross-hatched bars indicate the ARA- and/or ATZ-treated conditions. Data are presented as the mean ± SD of three independent experiments. * *p* < 0.05, ** *p* < 0.01, and *** *p* < 0.001 compared with no treatment or M1 + ARA.

The difference in UGT activity between M1 and M2 macrophages was further evident from the extent of glucuronidation of PGE_2_, an immunoregulatory lipid mediator [10]. Upon treatment with ARA, the precursor of PGE_2_ [26], comparable levels of free PGE_2_ were detected in both macrophage phenotypes (Figure 4A), while PGE_2_-glucuronide levels were higher in M1 compared with M2 macrophages (Figure 4B). Using ATZ as a UGT inhibitor [27], PGE_2_-glucuronide levels were decreased (Figure 4B), while free PGE_2_ levels were increased (Figure 4A) in both M1 and M2 macrophages, indicating the role of UGTs in the regulation of PGE_2_ levels in these macrophages. To further elucidate the role of UGTs in PGE_2_ metabolism in M1 macrophages, a time-course quantitation was performed. Following ATZ treatment, free PGE_2_ concentrations were higher at each time point than under ARA-only conditions (Figure 4C), whereas PGE_2_-glucuronide concentrations were lower (Figure 4D), indicating that UGTs contribute to the regulation of PGE_2_ metabolism.

### 3.4. Effect of UGT Inhibition on Pro-Inflammatory Markers in M1 Macrophages

**Figure 5:**
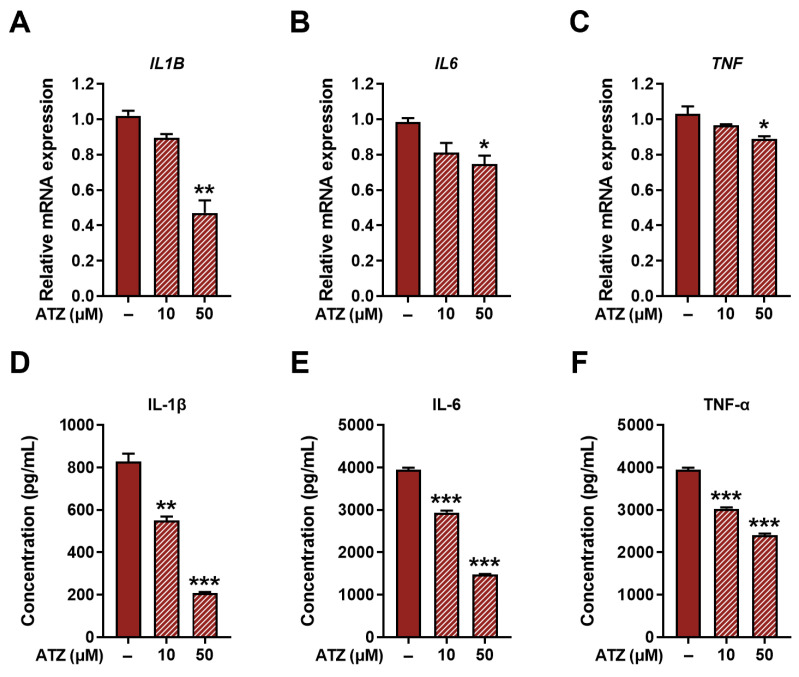
Impact of ATZ on production of pro-inflammatory cytokines in M1 macrophages. Relative mRNA expression levels of (**A**) *IL1B*, (**B**) *IL6*, and (**C**) *TNF* in M1 macrophages treated with the UGT inhibitor ATZ. Protein levels of (**D**) IL-1β, (**E**) IL-6, and (**F**) TNF-α in the culture supernatants of M1 macrophages following ATZ treatment. Cross-hatched bars indicate ATZ-treated conditions. Data are presented as the mean ± SD of three independent experiments. * *p* < 0.05, ** *p* < 0.01, and *** *p* < 0.001 compared with no treatment.

Finally, considering the association between PGE_2_ and pro-inflammatory responses, it was investigated whether UGT inhibition affects pro-inflammatory markers within M1 macrophages. Upon ATZ treatment, mRNA levels of pro-inflammatory cytokines, including IL-1β, IL-6, and TNF-α, were significantly reduced in M1 macrophages (Figure 5A–C). Consistent with these transcriptional changes, protein levels of IL-1β, IL-6, and TNF-α were also decreased following ATZ treatment (Figure 5D–F). These findings support a functional link between UGT-mediated PGE_2_ metabolism and the regulation of pro-inflammatory cytokine production in M1 macrophages.

**Figure 6:**
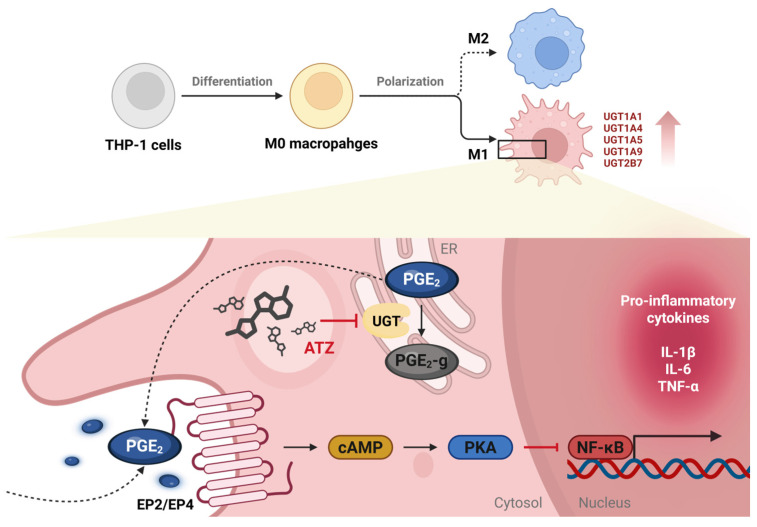
Schematic illustration of the proposed role of UGTs in regulating macrophage inflammatory responses. M1 macrophages exhibit increased expression of multiple UGT enzymes, which promotes the glucuronidation of PGE_2_. In its free form, PGE_2_ binds to EP2 or EP4 receptors, leading to an increase in intracellular cAMP levels and subsequent activation of PKA. Activated PKA suppresses NF-κB, thereby reducing the expression of pro-inflammatory cytokines. Inhibition of UGTs by ATZ suppresses PGE_2_ glucuronidation, resulting in increased levels of free PGE_2_ and reduced production of pro-inflammatory cytokines in M1 macrophages. cAMP, cyclic AMP; ER, endoplasmic reticulum; EP, prostaglandin E; PGE_2_, prostaglandin E_2_; PGE_2_-g, PGE_2_-glucuronide; PKA, protein kinase A; UGT, UDP-glucuronosyltransferase.

Taken together, these findings demonstrate that UGT inhibition attenuates the pro-inflammatory phenotype of M1 macrophages, in association with altered PGE_2_ metabolism and downstream inflammatory signaling, as illustrated in Figure 6.

## 4. Discussion

The present study provides novel evidence that M1 and M2 macrophages differ substantially in UGT expression, enzymatic activity, and downstream inflammatory signaling. Notably, M1 macrophages exhibited higher expression of UGT1A1, UGT1A4, UGT1A5, UGT1A9, and UGT2B7, accompanied by heightened glucuronidation activity, compared with M2 macrophages. Given that well-established immunosuppressive effects of PGE_2_ [28,29], the effects of differences in UGT-mediated PGE_2_ glucuronidation between M1 and M2 macrophages on inflammatory responses were therefore considered worthy of investigation. Enhanced UGT-mediated glucuronidation of PGE_2_ in M1 macrophages was associated with reduced levels of free PGE_2_ and increased production of pro-inflammatory cytokines such as IL-1β, IL-6, and TNF-α, which are known to reinforce inflammatory signaling and stabilize the M1 pro-inflammatory phenotype [30]. In this study, we identified distinct differences in UGT expression and enzymatic activity between M1 and M2 macrophages, suggesting that UGT enzyme system could be linked to macrophage inflammatory responses.

Tochigi et al. reported that the expression of UGT1A1 and UGT1A7 was increased in LPS-stimulated rat macrophages, thereby promoting the metabolism of toxic substrates [9]. Considering this, the mechanism proposed in the present study, the exacerbation of pro-inflammatory properties through UGT-mediated PGE_2_ glucuronidation in M1 macrophages, suggests that the influence of LPS/IFN-γ signaling cannot be entirely excluded. Furthermore, given the broad substrate spectrum of UGTs toward endogenous lipid mediators [7], the attenuation of pro-inflammatory cytokines observed following UGT inhibition may reflect integrated metabolic effects beyond PGE_2_, although the concurrent reduction in PGE_2_-glucuronide and pro-inflammatory cytokines supports a mechanistic association. It also remains to be determined whether UGT-mediated glucuronidation regulates other bioactive lipid mediators, including specialized pro-resolving lipid mediators such as resolvins, protectins, and maresins [31,32,33], thereby implicating UGT-mediated lipid metabolism in orchestrating the resolution phase of inflammation.

In experiments using pharmacological inhibitors, ATZ reduced glucuronidation of PGE_2_ while increasing levels of free PGE_2_, accompanied by decreased production and secretion of pro-inflammatory cytokines (Figure 6). To avoid potential confounding effects of diclofenac on prostaglandin synthesis [34], ATZ was employed to inhibit PGE_2_ glucuronidation. ATZ, known as a substrate and inhibitor CYP3A4 [35], has also been used as a UGT inhibitor in several studies [36,37]. Given that CYP3A4 is primarily involved in the metabolism of drugs or exogenous compounds [38] and is not known to be involved in lipid metabolism, potential off-target effects on PGE_2_ metabolism in the present study are likely to be minimal. Furthermore, as no highly selective chemical inhibitor of UGTs is currently available, ATZ was considered an appropriate agent for inhibiting UGT-mediated PGE_2_ glucuronidation. Nonetheless, given the inherent off-target effects of pharmacological inhibitors, genetic approaches using siRNA or CRISPR-Cas9 could further strengthen mechanistic specificity.

PGE_2_ dynamically exerts pro- or anti-inflammatory effects through engagement of distinct prostaglandin E (EP) receptor subtypes (EP1-EP4), depending on cellular and microenvironmental context. Engagement of EP2 and EP4 signals through Gα_s_ increased cellular cAMP levels and subsequent activation of protein kinase A (PKA). Activated PKA suppresses NF-κB signaling, thereby attenuating the production of pro-inflammatory cytokines [39]. Consistent with previous study reporting that predominant expression of EP2 and EP4 among four types of EP receptors in THP-1 cells [40], the elevated PGE_2_ levels observed following ATZ treatment are likely to preferentially activate EP2 and EP4, which may underline the reduction in pro-inflammatory cytokine production. However, as these findings were obtained from cell experiments under defined in vitro conditions, validation using macrophages derived from human in vivo samples would provide stronger evidence for the physiological relevance of this study.

Given the clinical observations that increased UGT2B17 expression in chronic lymphocyte leukemia correlates with heightened PGE_2_ glucuronidation and poor prognosis [14], this study underscores the potential clinical significance of UGT-mediated PGE_2_ glucuronidation. Notably, the tumor microenvironment is often enriched in PGE_2_, which induces the polarization of macrophages toward M2-like tumor-associated macrophages (TAMs) and promotes tumor progression through an immunosuppressive milieu. Accordingly, cyclooxygenase (COX) inhibitors have been investigated as anticancer agents to reduce PGE_2_ production [41]. However, such approaches broadly suppress prostanoid synthesis, potentially leading to off-target effects and undesirable systemic toxicity. In this context, targeting UGTs could serve as an alternative therapeutic strategy to control inflammatory responses by precisely regulating the availability of PGE_2_ and other endogenous lipid mediators.

Although a differentiation protocol previously used in this laboratory was employed, validation using a broader panel of canonical M1 and M2 markers enables clearer confirmation of macrophage polarization. In addition to pharmacological inhibition, complementary approaches such as siRNA-mediated knockdown of key UGT isoforms would enhance mechanistic evidence. Cytotoxicity following chemical treatment was assessed based on cellular morphology, as multiple previous studies using the same cell system have been conducted in this laboratory. However, additional quantitative analysis, such as MTT or CCK-8 assays, would provide more certainty regarding cellular conditions.

In summary, our findings reveal an unrecognized role for UGTs in modulating the balance between pro- and anti-inflammatory signaling in macrophages through the regulation of PGE_2_, thereby offering important insight into the functional distinctions between M1 and M2 phenotypes. Further investigation of UGT expression and activity during immune activation in vivo will be critical for defining the physiological relevance of UGT-mediated metabolism. Collectively, this study substantially advances the understanding of UGT biology, an area that has remained largely underappreciated in the context of macrophage function and inflammatory regulation.

## 5. Conclusions

In this study, we identified distinct patterns of UGT expression and enzymatic activity according to macrophages’ polarization state. Elevated UGT activity in M1 macrophages contributed to maintenance of the pro-inflammatory phenotype by limiting endogenous PGE_2_. Collectively, our results suggest that UGTs function not only as phase II metabolic enzymes but also as regulatory modulators of inflammatory signaling within macrophages. From a therapeutic perspective, selective modulation of UGT activity may represent an alternative strategy to conventional COX inhibition, as regulating PGE_2_ bioavailability could provide a microenvironment-based approach to controlling inflammatory responses. Further validation of these findings in in vivo models would be necessary to support potential clinical application.

## Data Availability

The original contributions presented in this study are included in the article and Supplementary Material. Further inquiries can be directed to the corresponding author.

## Supplementary Materials

The following supporting information can be downloaded at: https://www.mdpi.com/article/10.3390/jpm16030160/s1, Table S1: Primers for RT-PCR.

## Abbreviations

The following abbreviations are used in this manuscript:

ARA	Arachidonic acid
ATZ	Atazanavir
COX	Cyclooxygenase
IFN-γ	Interferon gamma
IL	Interleukin
LPS	Lipopolysaccharide
NSAID	Non-steroidal anti-inflammatory drug
PGE_2_	Prostaglandin E_2_
PMA	Phorbol 12-myristate 13-acetate
TNF-α	Tumor necrosis factor alpha
UDPGA	UDP-glucuronic acid
UGT	UDP-glucuronosyltransferase
