# Supplementary material for: Modulation of Macrophage Inflammatory Responses by UDP-Glucuronosyltransferase-Mediated PGE2 Glucuronidation
Source: J Pers Med. 2026 Mar 13;16(3):160. doi: 10.3390/jpm16030160 (PMC13028322; doi:10.3390/jpm16030160)
Supplement: Supplementary file 1 [file jpm-16-00160-s001.zip › jpm-4128388-supplementary.pdf]

Supplementary Materials

Table S1. Primers for RT-PCR

Gene	Orientation[a]	Sequence (5′→3′)
UGT1A1	F	CCTTGCCTCAGAATTCCTTC
	R	ATTGATCCCAAAGAGAAAACCAC
UGT1A3	F	CAGTGGTGGATATTCTCAGTC
	R	CCATGTTCTCCAGAAGCATTA
UGT1A4	F	ACGCTGGGCTACACTCAAGG
	R	TCTGAATTGGTCGTTAGTAACT
UGT1A5	F	ACAATATGTCTTTGATCATA
	R	AGAAACAGCATGGCAAAG
UGT1A6	F	AGAGAATTTCTGCAGGGGTTTT
	R	TTGGATTCTTTCAAAAGC
UGT1A7	F	TGGCTCGTGCAGGGTGGACTG
	R	TTCGCAATGGTGCCGTCCAGC
UGT1A8	F	GGTCTTCGCCAGGGGAATAG
	R	ATTGATCCCAAAGAGAAAACCAC
UGT1A9	F	GAGGAACATTTATTATGCCACCG
	R	GCAACAACCAAATTGATGTGTG
UGT1A10	F	CTCTTTCCTATGTCCCAATGA
	R	ATTGATCCCAAAGAGAAACCAC
UGT2B4	F	CATCTTCAGCTTCCATTTC
	R	TCCTTACAGAACTTTCTAAG
UGT2B7	F	AGTTGGAGAATTTCATCATGCAACAGA
	R	TCAGCCCAGCAGCTCACCACAGGG
UGT2B10	F	TGACATCGTTTTTGCAGATGCTTA
	R	CAGGTACGTAGGAAGGAGGGAA
UGT2B11	F	CTTCCATTCTTTTTGATCCCAATGAT
	R	GGAGACTGTACACAAACC
UGT2B15	F	GTGTTGGGAATATTATGACTACAGTAAC
	R	TCAGCCAGTAGCTCACCACAGGG
UGT2B17	F	GTGTTGGGAATATTCTGACTATAATATA
	R	CAGGTACATAGGAAGGAGGGAA
UGT2B28	F	ATCCCAATGACGCATTCACTCTTAAACTC
	R	CAGGTATGTAGGAAGGAGGGAA
ACTB	F	GGCGGCACCACCATGTACCCT
	R	AGGGGCCGGACTCGTCATACT

[a] F, forward; R, reverse. Gene specific primers were designed by using NCBI Primer blast tool, https://www.ncbi.nlm.nih.gov/tools/primer-blast.
